# Supplementary material for: Revealing Changes in Ovarian and Hemolymphatic Metabolites Using Widely Targeted Metabolomics between Newly Emerged and Laying Queens of Honeybee (Apis mellifera)
Source: Insects. 2024 Apr 11;15(4):263. doi: 10.3390/insects15040263 (PMC11050517; doi:10.3390/insects15040263)
Supplement: Supplementary file 1 [file insects-15-00263-s001.zip › Supplementary Materials/Supplementary Figure.pdf]

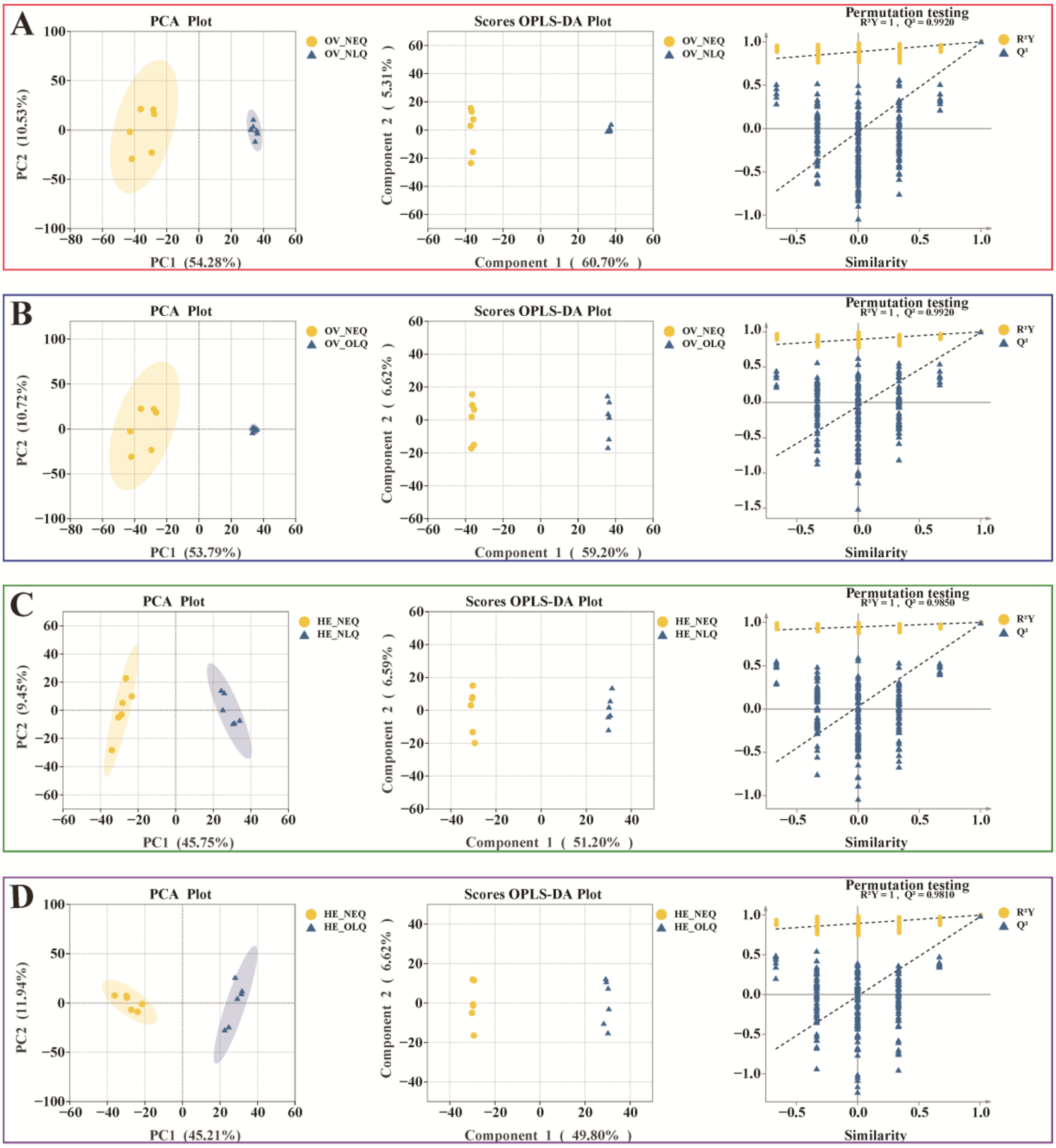

Figure S1: The PCA plots, the OPLS-DA score plots, and the OPLS-DA permutation test plots are in each group. (A) The group NLQ vs. NEQ in the ovary. (B) The group OLQ vs. NEQ in the ovary. (C) The group NLQ vs. NEQ in the hemolymph. (D) The group OLQ vs. NEQ in the hemolymph.
